# Quantitative evaluation of disease severity in connective tissue disease-associated interstitial lung disease by dual-energy computed tomography

**DOI:** 10.1186/s12931-022-01972-4

**Published:** 2022-03-05

**Authors:** Ling Chen, Min Zhu, Haiyan Lu, Ting Yang, Wanjiang Li, Yali Zhang, Qibing Xie, Zhenlin Li, Huajing Wan, Fengming Luo

**Affiliations:** 1 Department of Respiratory and Critical Care Medicine, West China Hospital, Sichuan University, No. 37 Guo Xue Xiang, Chengdu, 610041 China; 2 Laboratory of Pulmonary Immunology and Inflammation, Frontiers Science Center for Disease-Related Molecular Network, Sichuan University, No.2222 Xin Chuan Road, Chengdu, 610041 Sichuan China; 3 Department of Radiology, West China Hospital, Sichuan University, Chengdu, 610041 China; 4 Department of Rheumatology, West China Hospital, Sichuan University, Chengdu, 610041 China

**Keywords:** Connective tissue disease-associated interstitial lung disease (CTD-ILD), Dual-energy computed tomography (DECT), Lung volume (LV), Effective atomic number (Z_eff_), Monochromatic CT number (MCTN)

## Abstract

**Background:**

High-resolution computed tomography (HRCT) is recommended diagnosing and monitoring connective tissue disease-associated interstitial lung disease (CTD-ILD). Quantitative computed tomography has the potential to precisely assess the radiological severity of CTD-ILD, but has still been under study.

**Objective:**

To investigate whether dual-energy computed tomography (DECT), a novel quantitative technique, can be used for quantitative severity assessment in CTD-ILD.

**Methods:**

This cross sectional study recruited adult CTD-ILD patients who underwent DECT scans from the ICE study between October 2019 and November 2021. DECT parameters, including effective atomic number (Z_eff_), lung (lobe) volume, and monochromatic CT number (MCTN) of each lung lobe, were evaluated. CTD-ILD was classified into extensive CTD-ILD and limited CTD-ILD by staging algorithm using combined forced vital capacity (FVC)%predicted and total extent of ILD (TEI) on CT. Dyspnea, cough, and life quality were scored by Borg dyspnea score, Leicester cough questionnaire (LCQ), and short-form 36 health survey questionnaire (SF-36), respectively.

**Results:**

There was a total of 147 patients with DECT scans enrolled. Higher Z_eff_ value (3.104 vs 2.256, *p* < 0.001), higher MCTN (− 722.87 HU vs − 802.20 HU, *p* < 0.001), and lower lung volume (2309.51cm^3^ vs 3475.21cm^3^, *p* < 0.001) were found in extensive CTD-ILD compared with limited CTD-ILD. DECT parameters had significant moderate correlations with FVC%predicted (|r|= 0.542–0.667, *p* < 0.01), DLCO%predicted (|r|= 0.371–0.427, *p* < 0.01), and TEI (|r|= 0.485–0.742, *p* < 0.01). Receiver operating characteristic (ROC) analysis indicated MCTN averaged over the whole lung had the best performance for extensive CTD-ILD discrimination (AUC = 0.901, cut-off: − 762.30 HU, *p* < 0.001), with a sensitivity of 82.1% and a specificity of 85.4%. The Z_eff_ value was the independent risk factor for dyspnea (OR = 3.644, 95% CI: 1.846–7.192, p < 0.001) and cough (OR = 3.101, 95% CI: 1.528–6.294, p = 0.002), and lung volume significantly contributed to the mental component summary (MCS) in SF-36 (standardized β = 0.198, *p* < 0.05).

**Conclusions:**

DECT can be applied to evaluate the severity of CTD-ILD.

**Supplementary Information:**

The online version contains supplementary material available at 10.1186/s12931-022-01972-4.

## Background

Connective tissue disease-associated interstitial lung disease (CTD-ILD) is a predominant type of ILD [1], as well as a common and serious pulmonary complication of CTD [2, 3]. It has been reported to be the leading cause of significant morbidity and mortality of CTD [4], constituting 31.76% mortality of this disease [5]. The survival time of CTD-ILD patients was 1.7 times shorter than CTD patients without ILD [5]. The severity of ILD is usually associated with poor outcome and life quality of CTD-ILD patients [6, 7]. Therefore, accurate evaluation of ILD severity may provide evidence for the appropriate clinical decision in the diagnosing and monitoring of CTD-ILD patients.

Pulmonary function test (PFT) and high-resolution computed tomography (HRCT) have been still the two major tools to diagnose and evaluate CTD-ILD in clinical practices, rather than lung biopsy [8]. Declines of PFTs, such as forced vital capacity (FVC), vital capacity, and diffusing capacity of the lungs for carbon monoxide (DLCO), are predictors for exacerbations and mortality in ILD [9]. But the results of PFTs weakly reflect the extent of ILD [9] and could be confounded by the presence of comorbidities, such as pleural disease [10], chronic airway diseases, pulmonary hypertension, or anemia [11, 12]. Furthermore, given that the measures of PFT heavily rely on the cooperation of patients [13], PFT is not applicable to some severe patients. HRCT can define different radiological patterns and distribution of ILD abnormalities, which is fundamental to the diagnosis and monitoring of ILDs. Increased extent of fibrotic abnormalities, including honeycombing, reticulation, and traction bronchiectasis, were predictors of worse prognosis in ILD [14, 15]. However, most assessments in the above studies were visual or semi-quantitative analysis, which was subjective evaluation, and relied on experienced radiologists or pulmonologists [16]. Recently, there have been several quantitative assessment methods under investigation, like quantitative lung fibrosis (QLF) score [17], and computer-aided lung informatics for pathology evaluation and ratings (CALIPER) [18], but these methods require additional professional software and senior radiologists or pulmonologists. Therefore, it is worth exploring a more precise and convenient quantitative evaluation technology.

In conventional CT imaging, the only quantitative value we can obtain in the images is the CT number. It is a calculated value reflecting the X-ray attenuation coefficient in an image voxel, representing the combination of atomic number of the materials and their density. As such, different materials with different atomic numbers on different densities may generate same CT number. It is challenging for CT number to differentiate and classify different types of tissues**.** Dual-energy computed tomography (DECT) [19, 20] was introduced to overcome the shortcomings of conventional CT by generating multiple imaging parameters to differentiate and classify a mixture of different materials without additional dose or compromised image quality [21]. The imaging parameters in DECT include the effective atomic number (Z_eff_), monochromatic CT number (MCTN), and lung lobe volume, etc. The Z_eff_ value reflects the intrinsic composition of a material, allowing the application of atomic number to answer what the material is. It is independent on the photon energy and relates to the mix of elements that will be in the voxel. MCTN is the CT numbers generated from monochromatic images in DECT. The monochromatic image depicts how the imaged object would look when the X-ray source produced only X-ray photons at a single energy. This allows MCTN better represents the true attenuation of materials to X-ray beams. Lung lobe volume reflects lung compliance, which allows the evaluation of ventilation function. And DECT has been demonstrated to simultaneously provide the lung function information, as well as the anatomic information in pulmonary diseases [22]. Up to now, DECT has shown its clinical applications value in malignant lung lesions, chronic airway diseases, pulmonary embolism, and pulmonary ventilation abnormalities [25–28]. However, its value in interstitial lung disease is still unknown.

Considering the ability of DECT to structurally and functionally image lung lesions, we hypothesized that DECT could be applied in CTD-ILD severity assessment. To test this hypothesis, DECT parameters in different severity of CTD-ILD patients were evaluated, and the correlations of DECT parameters with the levels of PFTs, the extent of ILD, manifestations of respiratory symptoms (dyspnea and cough) and life quality levels were investigated, respectively.

## Material and methods

### Study design and subjects

This cross sectional study was designed to explore whether DECT parameters could reflect the severity of CTD-ILDs evaluated by CT, PFTs (FVC%predicted and DLCO%predicted), symptoms scales (dyspnea scored by Borg dyspnea score, cough scored by Leicester cough questionnaire (LCQ)), and life quality scale (36 items short form health survey (SF-36)). This study was approved by Ethics Committee on Biomedical Research (2020–216) at West China Hospital, and all the patients had written informed consent.

**Fig. 1:**
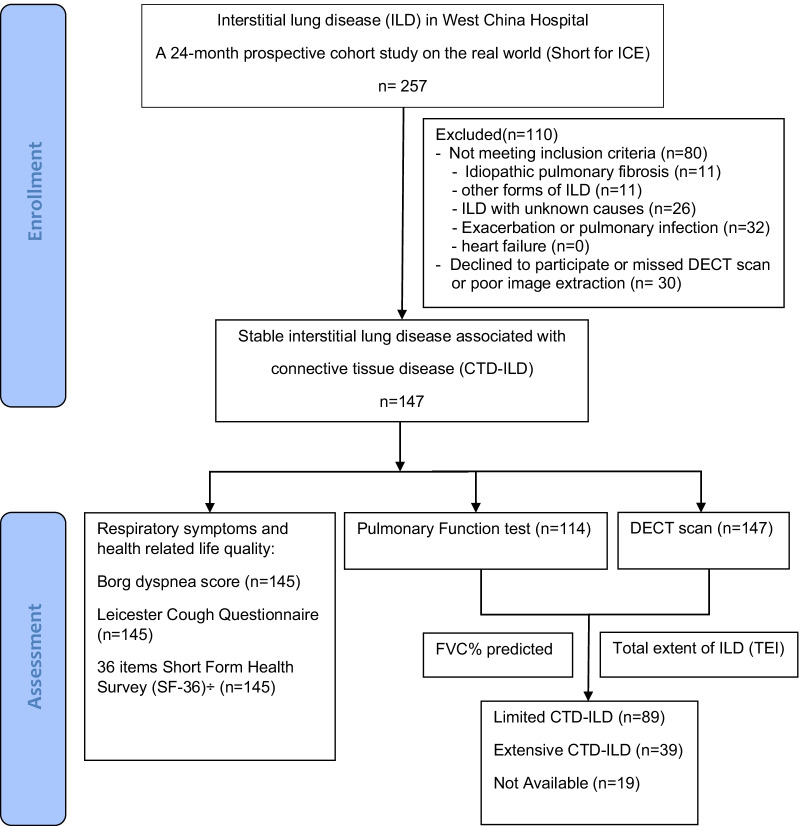
Cohort formation. Patients of stable CTD-ILD underwent the DECT with good image extraction were included. DECT Dual-energy computed tomography; FVC forced vital capacity

A total of 147 adult stable CTD-ILD patients with DECT scans were enrolled, followed up in ICE study (*I*nterstitial lung disease in West China Hospital: a 24-month prospective *C*ohort on the r*E*al world) between October 2019 and November 2021. The diagnosis of CTD-ILD was determined by the ILD multidisciplinary group with experts in radiology, pathology, rheumatology, and pulmonology according to CTD-ILD position statement from the Thoracic Society of Australia and New Zealand [2] and the official statement of the European Respiratory Society/American Thoracic Society for interstitial pneumonia with autoimmune features (IPAF) [30]. Exclusion criteria included idiopathic ILD, ILD for other causes (such as sarcoidosis, drug, toxicity, or PAP), acute exacerbation of interstitial lung disease according to the International Working Group Report of acute exacerbation of idiopathic pulmonary fibrosis [31], pulmonary infection, heart failure [32] to avoid the influence on the DECT parameters; refused consent; or missing DECT scan data. Figure 1 showed the process of patients screening. The lung images data of healthy people from a health checking program was also included in the stratification analysis.

### Data collection

The demographics and clinical characteristics were reviewed by the well-trained researchers, including basic information (age, sex, body mass index, smoking history), respiratory symptoms (dyspnea and cough), quality of life (SF-36, consisting of physical component summary (PCS) and mental component summary (MCS), common comorbid conditions (chronic obstructive pulmonary disease, coronary artery disease, diabetes mellitus, hypothyroidism, anemia, etc.). Dyspnea was classified into no dyspnea (Borg dyspnea score = 0), mild dyspnea (0 < Borg dyspnea score ≤ 2) and moderate to severe dyspnea (Borg dyspnea score ≥ 3). Cough was classified into no cough (LCQ = 0), mild cough (LCQ ≥ 17) and severe cough (0 < LCQ < 17) [33].

Pulmonary function test was conducted according to the recommendations of the American Thoracic Society/European Respiratory Society [34]. The combination of FVC%predicted and DLCO%predicted was classified into mild (FVC%predicted ≥ 80% and DLCO%predicted ≥ 80%), moderate (FVC%predicted > 50% and DLCO%predicted > 50% and not the mild cases), and severe (FVC%predicted ≤ 50% or DLCO%predicted ≤ 50%), respectively.

### Image acquisition with DECT

All patients were scanned on a 256-slice CT scanner (Revolution CT, GE Healthcare, Milwaukee WI, USA) without contrast injection according to standards set. The DECT was obtained with the following parameters: fast switching between tube voltages of 80 and 140 kV, automatic mA selection for obtaining a noise index (NI) of 15, the spiral pitch of 0.984, tube rotation time of 0.5 s, and detector width of 80 mm (128 × 0.625 mm). Images were reconstructed using a “detail” kernel with a slice thickness of 1.25 mm and a reconstruction interval of 0.625 mm.

**Fig. 2:**
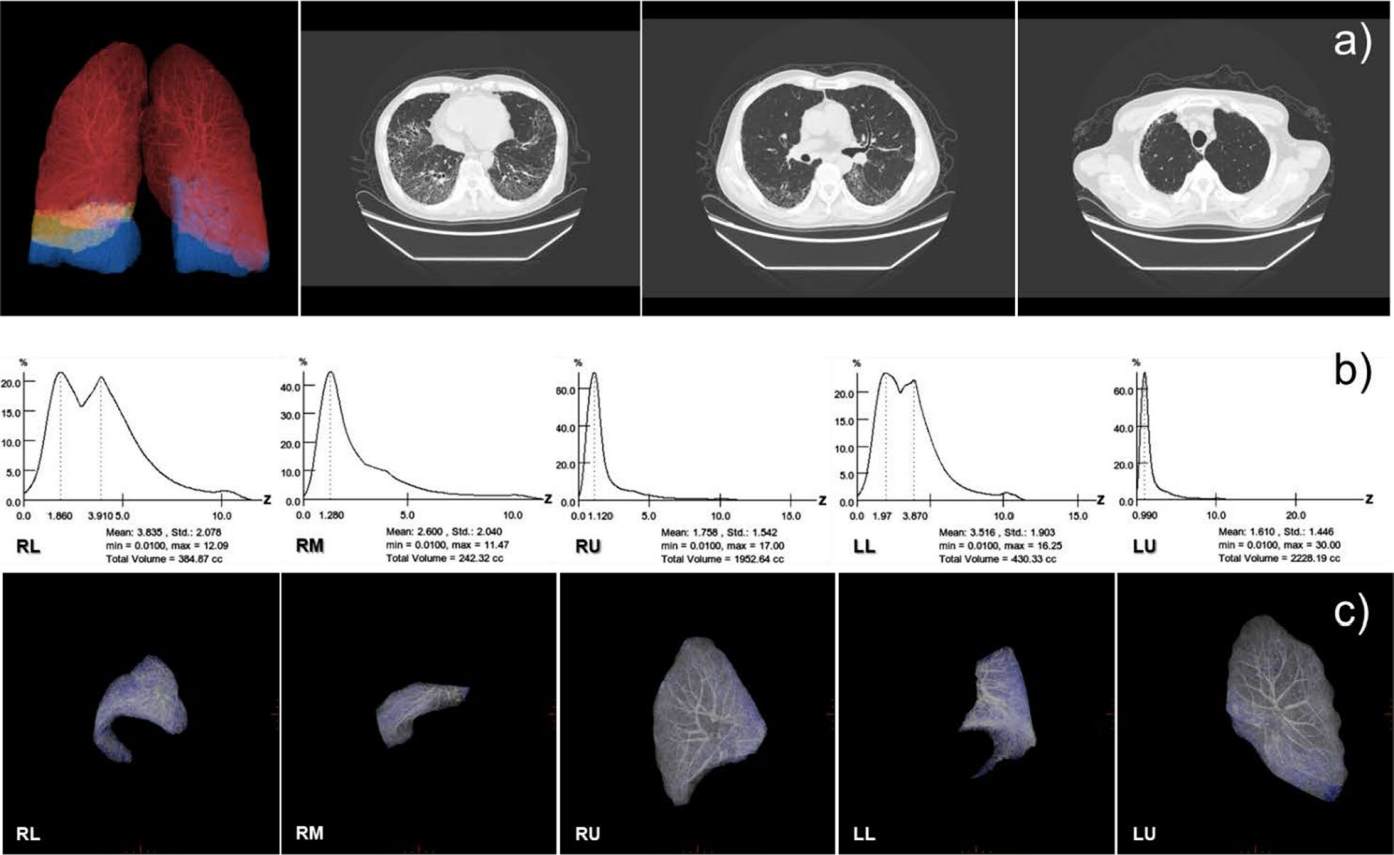
The component distribution histograms of DECT parameter in a 66-year-old man, diagnosed with interstitial lung disease associated with MCTD (PM/DM and Sjögren syndrome). Row **a** presents colored split lung lobes in 3D and axial computed tomographic images of the chest at the three levels. The histograms of Z_eff_ value were shown in Row **b**. Compared with each other, shape of the histogram in lower lung lobes with complex lesions was wider, the mean value was higher and the wave peaks are more than those of the middle and upper lung lobes. In RL, RM, RU, LL, LU order, the mean Z_eff_ value was 3.835, 2.600, 1.758, 3.516 and 1.610 respectively. Row **c** presented each lung lobe along with bronchovascular bundle in three-dimension (3D), and blue represents the interstitial lung abnormalities

The DECT images were transferred to an advanced workstation (ADW4.7) equipped with the Gemstone Spectral Imaging (GSI) for post-processing. The data processing step included lung extraction and segmentation with identification and extraction of central airways and manual segmentation of the anatomical lobes in the thoracic analysis software. The segmentation of the lung was achieved into the right upper lobe (RU), right middle lobe (RM), right lower lobe (RL), left upper lobe (LU), left lower lobe (LL) using an adaptive density-based morphological approach. The corresponding DECT parameters of each lung lobe were achieved according to the extracted histogram, such as lung lobe volume, the mean, minimum, maximum values of the effective atomic number (Z_eff_), and the monochromatic CT number (MCTN) at the 70 keV energy level (Fig. 2).

### The extent of ILD

The extent of ILD was visually scored at five levels (origin of great vessels, the main carina, the pulmonary venous confluence, halfway between the third and fifth sections, and immediately above the right hemidiaphragm), and the total extent of ILD (TEI) on DECT was calculated as the mean of the five scores, according to the previous ILD staging system. The extent of the abnormalities was estimated to be the nearest 5% in each section. 20% was selected to be the TEI threshold of severe, and TEI > 20% was proved to be the powerful predictor of mortality. All semi-quantitative image evaluations of ILD were performed by two independent readers (LC with 7 years, TY with 12 years of experience in thoracic CT diagnosis). Discordances were settled by consensus.

### Severity assessment of CTD-ILD

Limited/extensive staging system combined TEI and PFTs has greater mortality prognostic value compared with either TEI or PFT [36], and associates with the decline in FVC [37], so it is widely recommended to assess the severity of CTD-ILD. According to this staging system, TEI on CT is the preliminary analysis to identify cases readily classifiable into limited disease (TEI ≤ 10%) or extensive disease (TEI > 30%). For indeterminate TEI defined as TEI ranging from 10 to 30%, FVC%predicted with cut-off value 70% is combined. Thus, the CTD-ILD was staged as limited disease (TEI ≤ 10%, or indeterminate TEI with FVC%predicted ≥ 70%), or extensive disease (TEI > 30%, or indeterminate TEI with FVC%predicted < 70%), which represented the mild, or severe CTD-ILD, respectively.

### Statistical analysis

Continuous variables were presented as means ± SD or median (range). Count variables were presented as frequency or percentage. Differences were analyzed using the student’s t-test or ANOVA for continuous variables (LSD test was used for multiple comparisons), or chi-square test for count variables. Pearson correlation coefficients (r values) or Spearman correlation coefficients was used to quantify the correlation. Binary logistic regression was performed to study the association between DECT parameters and onset of dyspnea or cough symptom. Linear regression analysis was performed to determine the prediction of DECT parameters to the SF-36 in CTD-ILD patients. Receiver operating characteristics (ROC) analysis was performed to determine the area under the curve (AUC) for the diagnostic performance of DECT to differentiate the extensive from limited CTD-ILD. All data were presented for statistical analysis using SPSS 20.0 (IBM, Armonk, NY, USA). Differences corresponding to *p* < 0.05 were considered significant.

## Results

### Demographic and clinical characteristics

A total of 147 adult stable CTD-ILD patients with DECT scans were enrolled (Fig. 1), with 102 specific CTD (SCTD)-ILD, 30 IPAF, and 15 mixed CTD (MCTD)-ILD (Table 1). They were female predominant (89.8%), with a mean age of 50.10 ± 9.64 years old and BMI 23.10 ± 3.29 kg/m^2^, 11.6% with a smoking history, and 59.2% with comorbid conditions. Mean FVC%predicted was 89.59% ± 22.94%, and median DLCO%predicted was 74.55% (ranged from 58.13% to 84.48%). Combined with semi-quantitative chest CT assessment and FVC% predicted, 89 (60.5%) CTD-ILD patients were segregated into limited CTD-ILD, 39 (26.5%) extensive CTD-ILD and 19 (13.0%) unclassifiable for unavailable data. In available Borg dyspnea scores and LCQ scores, 69 (47.0%) patients had dyspnea, 64 (43.5%) had cough. The median PCS score of SF-36 was 51.80, ranged from 45.28 to 56.99, and the median MCS score of SF-36 was 52.33, ranged from 43.97 to 57.53.

**Table 1:** Connective tissue disease associated interstitial lung disease (CTD-ILD) cohort demographic and clinical characteristics

Demographic and clinical characteristics	Mean ± SD or N (%) or Median (range)
Age (year)	50.10 ± 9.64
Female, No. (%)	132 (89.8)
BMI (kg/m^2^) ^§^	23.10 ± 3.29
Ethnicity (Han nationality), No. (%)	136 (92.5)
Former/current smoker, No. (%)	17 (11.6)
Comorbidity, No. (%)	87 (59.2)
Type of CTD	
Specific CTD (SCTD)	102 (69.4)
Systemic sclerosis/scleroderma (SSc)	33 (22.4)
Polymyositis/dermatomyositis (PM/DM)	39 (26.5)
Sjögren Syndrome (SjS)	10 (6.8)
Rheumatoid Arthritis (RA)	11 (7.5)
Systemic lupus erythematosus (SLE)	9 (6.1)
Undifferentiated CTD (UCTD)	30 (20.4)
Mixed CTD (MCTD)	15 (10.2)
Dyspnea (Borg dyspnea score)	
No dyspnea	76 (51.7)
Mild dyspnea	53 (36.1)
Moderate to Severe dyspnea	16 (10.9)
NA	2 (1.4)
Cough (LCQ)	
No cough	81 (55.1)
Mild cough	39 (26.5)
Severe cough	25 (17.0)
NA	2 (1.4)
Pulmonary Function Test ^¶^	
FVC% predicted ^¶^	89.59 ± 22.94
FEV_1_%predicted ^¶^	87.50 ± 21.14
TLC%predicted ^¶^	85.80 ± 19.40
DLCO% predicted^+^	74.55 (58.13, 84.48)
Severity of CTD-ILD	
Limited CTD-ILD	89 (60.5)
Extensive CTD-ILD	39 (26.5)
NA	19 (13.0)
SF-36 ^λ^ (range 0–100)	
Physical component summary (PCS) score	51.80 (45.28, 56.99)
Mental component summary (MCS) score	52.33 (43.97, 57.53)

Data are presented as mean ± SD or n (%) or Median (range)

Total-No. = 147, unless otherwise stated; §: N = 146; λ: N = 145; ¶: N = 114; + : N = 112

*BMI* body mass index, *CTD* connective tissue disease, *ILD* interstitial lung disease, *LCQ* Leicester cough questionnaire, *FVC* forced vital capacity, *FEV*_1_ forced expiratory volume in one second, *TLC* total lung capacity, *DLCO* diffusion capacity of the lung for carbon monoxide, *SF-36* 36 item short form health survey, *NA* not available

### DECT parameters in different severity of CTD-ILDs

Differences of DECT parameters in different severity of CTD-ILDs were analyzed. According to the limited/extensive staging system combined TEI and PFTs, the extensive CTD-ILD patients (TEI > 30%, or indeterminate TEI with FVC%predicted < 70%) were shown to have significantly lower volume (2309.51 cm^3^ vs 3475.21 cm^3^, *p* < 0.001), higher Z_eff_ value (3.104 vs 2.256, *p* < 0.001), and higher MCTN (− 722.87 HU vs -802.20 HU, *p* < 0.001) in the whole lung, as well as each lung lobe (Table 2), when compared with limited CTD-ILDs (TEI ≤ 10%, or indeterminate TEI with FVC%predicted ≥ 70%). According to the classification of TEI or PFT respectively, the severe CTD-ILD patients (TEI > 20% for extent; FVC%predicted ≤ 50% or DLCO%predicted ≤ 50% for PFT) also had significant lower volume, higher Z_eff_ value, and higher MCTN compared with moderate and mild patients (TEI ≤ 20% for extent; FVC%predicted > 50% and DLCO%predicted > 50% for PFT) (Additional file 1: Table S1 and S2). Bilateral lower lungs of extensive ILD had the significantly smallest lobe volume, highest Z_eff_ value and MCTN when compared to those of limited ILD and normal lung images. And the lobe volumes and absolute values of MCTN in bilateral lower lung lobes decreased as the severity increased (*p* for linear trend < 0.01) (Additional file 1: Table S3).

**Table 2:** Comparison the chest DECT parameters between limited CTD-ILD and extensive CTD-ILD

	Limited CTD-ILD	Extensive CTD-ILD	t /χ^2^	*p*
Total-No	89	39		
Age (year)	49.44 ± 9.27	51.64 ± 11.55	− 1.146	*.254*
BMI (kg/m^2^)	23.49 ± 3.09	22.53 ± 3.64	1.515	*.132*
Female, No. (%)	78 (87.6%)	38 (97.4%)	3.062	*.080*
Han nationality, No. (%)	86 (96.6%)	35 (89.7%)	2.487	*.115*
Former/current smoker, No. (%)	14 (15.7%)	0 (0)	6.888	***.009***
Comorbidity, No. (%)	52 (58.4%)	24 (61.5%)	0.109	*.741*
Lung volume (cm^3^)	3475.21 ± 971.62	2309.51 ± 668.98	7.844	***.000***
V_RL	607.08 ± 275.18	278.39 ± 136.74	9.012	***.000***
V_RM	377.19 ± 138.00	287.47 ± 144.82	3.335	***.001***
V_RU	901.38 ± 249.48	742.69 ± 262.74	3.259	***.001***
V_LL	548.31 ± 255.13	254.51 ± 120.13	8.853	***.000***
V_LU	1041.24 ± 284.10	746.45 ± 316.93	5.215	***.000***
Average Z_eff_ value	2.256 ± 0.435	3.104 ± 0.569	− 8.311	***.000***
Z_RL	2.682 ± 0.653	3.828 ± 0.813	− 8.470	***.000***
Z_RM	2.018 ± 0.382	2.833 ± 0.692	− 6.906	***.000***
Z_RU	1.911 ± 0.327	2.501 ± 0.567	− 6.071	***.000***
Z_LL	2.706 ± 0.663	3.792 ± 0.698	− 8.398	***.000***
Z_LU	1.961 ± 0.334	2.566 ± 0.629	− 5.664	***.000***
Average MCTN (HU)	− 802.20 ± 40.27	− 722.87 ± 52.83	− 8.373	***.000***
MCTN_RL	− 760.08 ± 63.10	− 654.62 ± 75.83	− 8.173	***.000***
MCTN_RM	− 825.56 ± 35.10	− 749.33 ± 61.82	− 7.209	***.000***
MCTN_RU	− 836.07 ± 29.22	− 781.03 ± 54.10	− 5.982	***.000***
MCTN_LL	− 757.79 ± 63.46	− 654.72 ± 69.34	− 8.221	***.000***
MCTN_LU	− 831.51 ± 28.42	− 774.64 ± 60.19	− 5.631	***.000***

Total-No. = 128

*DECT* dual-energy computed tomography, *CTD-ILD* connective tissue disease-associated interstitial lung disease, *BMI* body mass index, *V_RL* volume of right lower lung lobe, *V_RM* volume of right middle lung lobe, *V_RU* volume of right upper lung lobe, *V_LL* volume of left lower lung lobe, *V_LU* volume of left upper lung lobe, *Z_RL* Z_eff_ value of right lower lung lobe, *Z_RM* Z_eff_ value of right middle lung lobe, *Z_RU* Z_eff_ value of right upper lung lobe, *Z_LL* Z_eff_ value of left lower lung lobe, *Z_LU* Z_eff_ value of left upper lung lobe, *MCTN* monochromatic CT number at 70 keV, *MCTN_RL* monochromatic CT number of right lower lung lobe, *MCTN_RM* monochromatic CT number of right middle lung lobe, *MCTN_RU* monochromatic CT number of right upper lung lobe, MCTN_LL monochromatic CT number of left lower lung lobe, *MCTN_LU* monochromatic CT number of left upper lung lobe

**Fig. 3:**
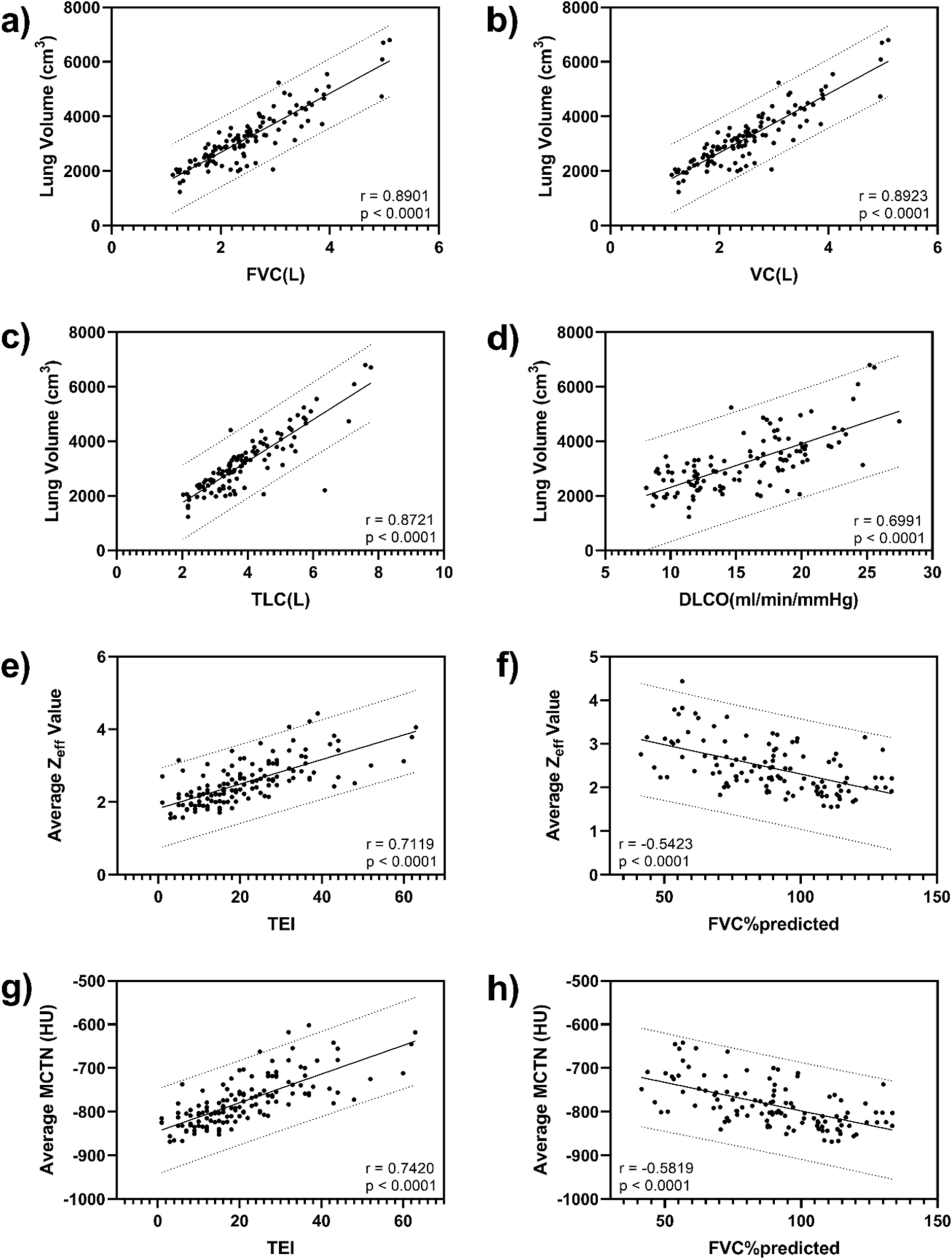
DECT parameters correlation with PFT findings and TEI. It was shown the lung volume highly correlated with FVC, VC, TLC and DLCO respectively in **a**–**d**. It was shown the moderate to high correlation of Z_eff_ value (**e**, **f**) and MCTN (**g**, **h**) with TEI and FVC%predicted respectively. Lines show estimated regression (solid line) and 95% confidence interval (dotted line)

The correlations of DECT parameters in whole lung and each lung lobe with PFT and semi-quantitative chest CT assessment were summarized in Additional file 1: Table S4. Figure 3 showed the LV positively correlated with VC (r = 0.8923, *p* < 0.0001), TLC (r = 0.8721, *p* < 0.0001), FVC (r = 0.8901, *p* < 0.0001) and DLCO (r = 0.6991, *p* < 0.0001), meanwhile Z_eff_ value and MCTN negatively correlated with FVC%predicted (r =  − 0.5423, *p* < 0.0001 for Z_eff_ value; r =  − 0.5819, *p* < 0.0001 for MCTN). The Z_eff_ value and MCTN showed a significantly high positive correlation with TEI (r = 0.7119, *p* < 0.01 for Z_eff_ value; r = 0.7420, *p* < 0.001 for MCTN).

**Fig. 4:**
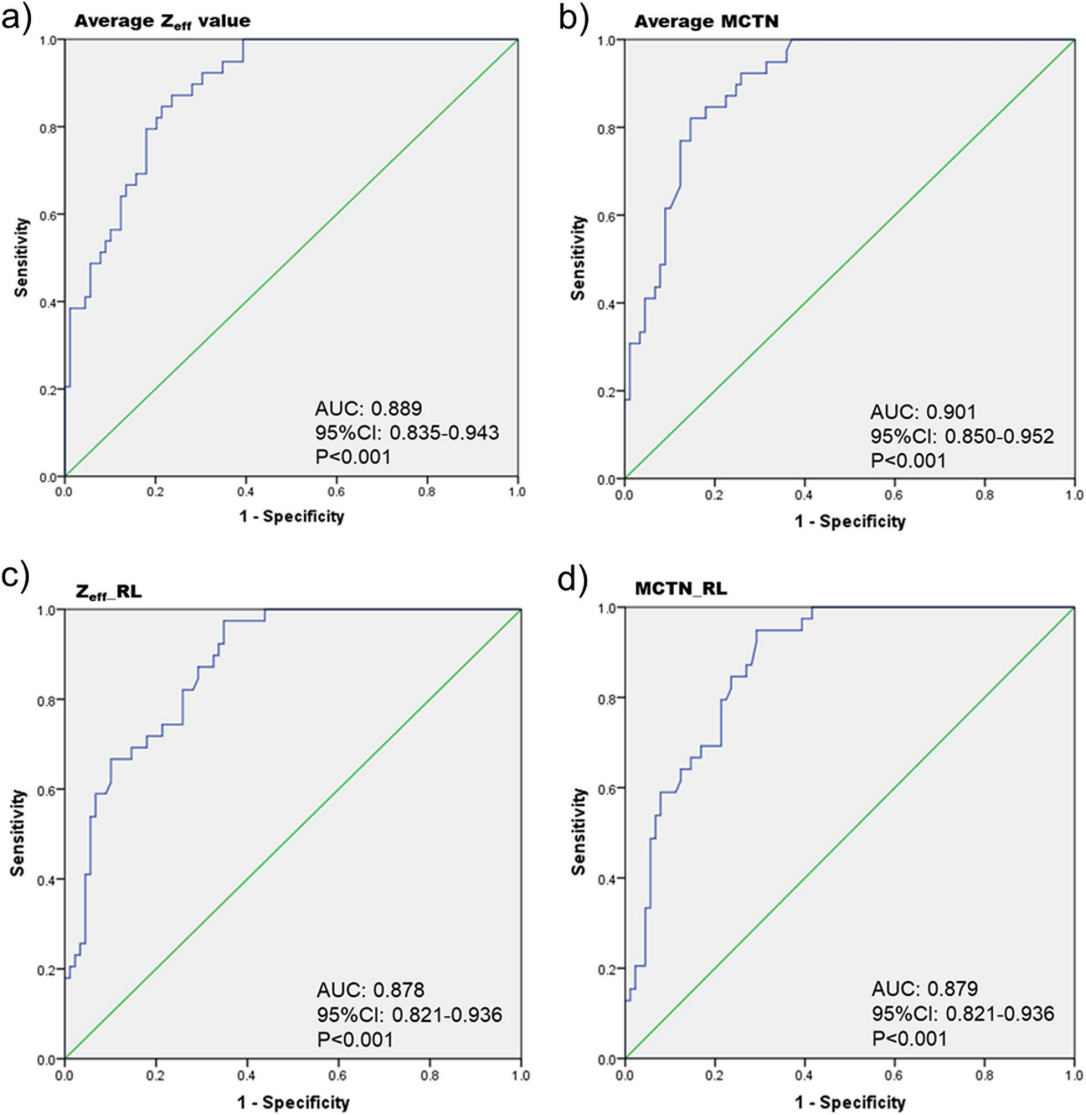
Receiver operating characteristic curve to demonstrate the optimal cutoff value of DECT parameters to detect presence of extensive disease of CTD-ILD. The optimal cut-off value was 2.510, 2.775, − 762.30 HU and − 746.50 HU for average Z_eff_ value, Z_eff__RL, average MCTN and MCTN_RL, respectively. AUC area under the curve, CI confidence interval, Z_RL Z_eff_ value of right lower lung lobe, MCTN_RL monochromatic CT number of right lower lung lobe. Average Z_eff_ value Z_eff_ value averaged over the whole lung, Average MCTN monochromatic CT number at 70 keV averaged over the whole lung

Among DECT parameters significantly differentiating extensive from limited CTD-ILDs, the Z_eff_ value and MCTN averaged over the whole lung, and those at middle lobe and lower lobes had higher AUCs (ranged from 0.864 to 0.901, *p* < 0.001) than lung (lobe) volume or those of upper lobes, shown in Fig. 4 and Additional file 1: Table S5. And the MCTN averaged over the whole lung (cutoff value = − 762.30 HU) achieved the highest AUC (0.901, 95%CI: 0.850–0.952), with a sensitivity of 82.1% and a specificity of 85.4%, to detect extensive CTD-ILD.

### DECT parameters’ performance in different symptoms of CTD-ILDs

The CTD-ILD patients with both dyspnea and cough had significantly smallest total lung volume (*p* = 0.003), highest Z_eff_ value * (p* = 0.000), and highest MCTN (*p* = 0.000) when compared with no respiratory symptom and one respiratory symptom, shown in Table 3. The similar trends were also shown in patients with severe dyspnea (Additional file 1: Table S6). Univariate analysis suggested that Z_eff_ value and MCTN were the risk factors for dyspnea and cough. MCTN was excluded in the multivariate analysis due to the significant collinearity with Z_eff_ value. The multivariate analysis suggested that Z_eff_ value was the independent risk factor for dyspnea (OR = 3.644, 95% CI: 1.846–7.192, *p* = 0.000) and cough (OR = 3.101, 95% CI: 1.528–6.294, *p* = 0. 002). In the same way, the multivariate analysis included MCTN was performed with Z_eff_ value excluded (Additional file 1: Tables S10 and S11, Tables 4, 5).

**Table 3:** Comparison the DECT parameters according to respiratory symptoms (dyspnea and cough based on Borg dyspnea score and LCQ)

	No symptom	Dyspnea or Cough	Dyspnea & Cough	F/χ2	*P* value	*P* value		
						0 *VS* 1	0 *VS* 2	1 *VS* 2
Total-No	49	59	37					
Age (y)	51.88 ± 9.15	49.95 ± 8.45	47.84 ± 11.33	1.918	*.151*			
BMI (kg/m^2^)	22.84 ± 2.90	23.22 ± 3.43	23.16 ± 3.60	0.196	*.822*			
Female (%)	42 (85.7)	52 (88.1)	36 (97.3)	3.297	*.192*			
Han nationality (%)	44 (89.8)	54 (91.5)	36 (97.3)	1.746	*.444*			
Former/current smoker (%)	6 (12.2)	10 (16.9)	1 (2.7)	4.479	*.107*			
Comorbidity (%)	25 (51.0)	40 (67.8)	20 (54.1)	3.533	*.171*			
Lung Volume (cm^3^)	3276.91 ± 901.49	3180.67 ± 1023.01	2587.13 ± 937.77	6.182	***.003***	*.605*	***.001***	***.004***
V_RL	536.65 ± 270.32	524.26 ± 277.83	360.07 ± 248.04	5.561	***.005***	*.811*	***.003***	***.004***
V_RM	344.79 ± 127.27	359.11 ± 147.82	331.00 ± 152.42	0.452	*.637*	*.604*	*.658*	*.348*
V_RU	892.29 ± 272.61	856.15 ± 232.68	779.98 ± 253.04	2.136	*.122*	*.459*	***.043***	*.152*
V_LL	500.43 ± 243.27	466.75 ± 50.63	313.56 ± 237.36	6.777	***.002***	*.478*	***.001***	***.003***
V_LU	1002.76 ± 293.67	974.40 ± 293.11	802.52 ± 317.40	5.358	***.006***	*.625*	***.003***	***.007***
Average Z_eff_ value	2.375 ± 0.425	2.398 ± 0.581	2.914 ± 0.629	12.819	***.000***	*.830*	***.000***	***.000***
Z_RL	2.886 ± 0.720	2.852 ± 0.790	3.603 ± 0.896	11.778	***.000***	*.829*	***.000***	***.000***
Z_RM	2.141 ± 0.349	2.174 ± 0.627	2.557 ± 0.691	6.584	***.002***	*.766*	***.001***	***.002***
Z_RU	1.957 ± 0.269	2.015 ± 0.504	2.350 ± 0.524	9.140	***.000***	*.506*	***.000***	***.000***
Z_LL	2.879 ± 0.720	2.881 ± 0.716	3.612 ± 0.833	13.184	***.000***	*.992*	***.000***	***.000***
Z_LU	2.011 ± 0.299	2.067 ± 0.474	2.450 ± 0.618	9.796	***.000***	*.542*	***.000***	***.000***
Average MCTN (HU)	− 790.51 ± 40.61	− 788.55 ± 53.41	− 740.97 ± 59.80	11.904	***.000***	*.843*	***.000***	***.000***
MCTN_RL	− 740.12 ± 71.29	− 744.80 ± 74.51	− 674.32 ± 83.27	11.260	***.000***	*.750*	***.000***	***.000***
MCTN_RM	− 813.31 ± 34.47	− 810.46 ± 57.87	− 776.43 ± 61.91	6.147	***.003***	*.779*	***.002***	***.002***
MCTN_RU	− 831.61 ± 24.88	− 826.00 ± 46.11	− 795.73 ± 50.54	8.541	***.000***	*.486*	***.000***	***.001***
MCTN_LL	− 741.27 ± 68.51	− 740.63 ± 66.50	− 671.70 ± 85.87	12.545	***.000***	*.964*	***.000***	***.000***
MCTN_LU	− 826.24 ± 27.76	− 820.85 ± 43.03	− 786.65 ± 58.80	9.033	***.000***	*.522*	***.000***	***.000***

0 = no respiratory symptom, 1 = with dyspnea or cough, 2 = with dyspnea and cough

Total-No. = 145

*DECT* dual-energy computed tomography, *LCQ* Leicester cough questionnaire, *CTD-ILD* connective tissue disease-associated interstitial lung disease, BMI body mass index, *V_RL* volume of right lower lung lobe, *V_RM* volume of right middle lung lobe, *V_RU* volume of right upper lung lobe, *V_LL* volume of left lower lung lobe, *V_LU* volume of left upper lung lobe, *Z_RL* Z_eff_ value of right lower lung lobe, *Z_RM* Z_eff_ value of right middle lung lobe, *Z_RU* Z_eff_ value of right upper lung lobe, *Z_LL* Z_eff_ value of left lower lung lobe, *Z_LU* Z_eff_ value of left upper lung lobe, *MCTN* monochromatic CT number at 70 keV, MCTN_RL monochromatic CT number of right lower lung lobe, *MCTN_RM* monochromatic CT number of right middle lung lobe, *MCTN_RU* monochromatic CT number of right upper lung lobe, *MCTN_LL* monochromatic CT number of left lower lung lobe, *MCTN_LU* monochromatic CT number of left upper lung lobe

**Table 4:** Binary logistic regression analysis of univariate analysis and multivariate analysis with dyspnea of CTD-ILD patient

	Univariate Analysis					Multivariate Analysis				
	β value	SE	Wals	OR (95%CI)	*P*	β value	SE	Wals	OR (95%CI)	*P*
Age (y)	− 0.020	0.018	1.328	0.980 (0.946–1.014)	*.249*	− 0.042	0.020	4.522	0.959 (0.923–0.997)	***.033***
Gender (male/female)	1.417	0.669	4.488	4.125 (1.112–15.304)	***.034***					
Han nationality	0.093	0.630	0.022	1.097 (0.319–3.770)	*.883*					
BMI (kg/m^2^)	0.063	0.051	1.516	1.065 (0.963–1.179)	*.218*					
Former/current smoke	− 0.875	0.561	2.436	0.417 (0.139–1.251)	*.119*					
Comorbidity	0.063	0.338	0.035	1.065 (0.549–2.065)	*.852*					
Lung volume (L)	− 0.461	0.188	5.982	0.631 (0.436–0.913)	***.014***					
Average Z_eff_ value	1.096	0.325	11.379	2.991 (1.583–5.653)	***.001***	1.293	.347	13.895	3.644 (1.846–7.192)	***.000***
Average MCTN (HU)	0.011	0.003	10.888	1.012 (1.005–1.018)	***.001***					
Type of CTD			2.398		*.302*					
SCTD vs UCTD	0.507	0.428	1.398	1.660 (0.717–3.843)	*.237*					
MCTD vs UCTD	0.952	0.649	2.151	2.591 (0.726–9.246)	*.142*					

Total, No. = 145, significance with p < 0.05

*CTD-ILD* connective tissue disease associated interstitial lung disease, *BMI* body mass index, *MCTN* monochromatic CT number at 70 keV, *CTD* connective tissue disease, *SCTD* specific connective tissue disease, *UCTD* undifferentiated connective tissue disease, *MCTD* mixed connective tissue disease

**Table 5:** Binary logistic regression analysis of univariate analysis and multivariate analysis with cough of CTD-ILD patient

	Univariate Analysis					Multivariate Analysis				
	β value	SE	Wals	OR (95%CI)	*P*	β value	SE	Wals	OR (95%CI)	*P*
Age (y)	-0.033	0.018	3.316	0.967 (0.933–1.003)	*.069*	-0.075	0.023	10.462	0.927 (0.886–0.971)	***.001***
Gender (male/female)	0.189	0.556	0.116	1.208 (0.407–3.592)	*.733*					
Han nationality	1.355	0.801	2.862	3.875 (0.807–18.614)	*.091*	1.499	0.864	3.010	4.479 (0.823–24.365)	*.083*
BMI (kg/m^2^)	-0.025	0.051	0.238	0.975 (0.882–1.078)	*.626*					
Former/current smoke	-0.137	0.524	0.068	0.872 (0.312–2.435)	*.794*					
Comorbidity	0.171	0.341	0.253	1.187 (0.609–2.314)	*.615*					
Lung volume (L)	-0.401	0.187	4.595	0.670 (0.464–0.966)	***.032***					
Average Z_eff_ value	0.771	0.302	6.504	2.161 (1.195–3.907)	***.011***	1.132	0.361	9.826	3.101 (1.528- 6.294)	***.002***
Average MCTN (HU)	0.008	0.003	6.318	1.008 (1.002–1.015)	***.012***					
Type of CTD			4.909		*.086*			10.637		***.005***
SCTD vs UCTD	-0.728	0.424	2.947	0.483 (0.210–1.109)	*.086*	-1.402	0.515	7.420	0.246 (0.090–0.675)	***.006***
MCTD vs UCTD)	-1.417	0.693	4.185	0.242 (0.062–0.942)	***.041***	-2.490	0.825	9.101	0.083 (0.016–0.418)	***.003***

Total, No. = 145, significance with p < 0.05

*CTD-ILD* connective tissue disease associated interstitial lung disease, *BMI* body mass index, *MCTN* monochromatic CT Number at 70 keV, *CTD* connective tissue disease,*SCTD* specific connective tissue disease,  *UCTD* undifferentiated connective tissue disease, *MCTD* mixed connective tissue disease

### DECT parameters contribute to the life quality of CTD-ILDs

The SF-36 health survey is an approach to measure life quality, and recommended for CTD-ILD patients. It was shown in our study that DECT parameters (LV, average Z_eff_ value, and average MCTN) significantly correlated with PCS score of SF-36 (*p* < 0.05, Additional file 1: Table S4), with |r| ranging from 0.231 to 0.283. The linear regression models showed that LV (standardized β = 0.198, *p* = 0.012) significantly contributed to the mental component summary (MCS) adjusted for age, gender, BMI, smoke, type of CTD and respiratory symptoms (Additional file 1: Table S9).

## Discussion

To our knowledge, this is the first research to evaluate the application value of unenhanced DECT with GSI in CTD-ILD patients. In this study, we showed that DECT parameters could reflect the clinical severity of CTD-ILD patients: increased severity of CTD-ILD assessed by symptoms, CT, and PFT significantly associated with reduced LV, elevated Z_eff_ value and increased MCTN. Z_eff_ value and MCTN had robust differentiation capacity to detect extensive CTD-ILD from limited CTD-ILD. Additionally, higher Z_eff_ value was the independent risk factor for dyspnea and cough in CTD-ILD patients. Lung volume reduction could reflect the CTD-ILD patients’ life quality.

In our study, DECT parameters could achieve excellent performance in terms of differentiating extensive from limited CTD-ILDs. The pathological basis of interstitial lung disease was pulmonary alveolar unit inflammation or interstitial fibrosis, leading to increasing mixture of various cells and intercellular matrix [39]. The interplay of multiple cell types, their cellular components, and intercellular matrix involved in the pathomechanism defines the diseases pattern, extent, function and severity of CTD-ILD. Z_eff_ and MCTN could represent the characteristics of the composite atoms for the mixture of various cells, materials or compounds in lesions [26], reflecting the severity of diseases. Lung fibrosis, including CTD-ILD, is characterized by restricted lung expansion [40]. In present study, DECT parameter lung volume highly correlated with VC, TLC and FVC: the lung volume reduced, accompanied by a reduction of VC, TLC and FVC in severe CTD-ILD patients, indicating restricted ventilatory defect [40], which might due to the reduction in lung compliance. Meanwhile, LV performed better than Z_eff_ value and MCTN in mental health evaluation. Therefore, DECT parameters exhibited complementary evaluation capacity of CTD-ILD.

It is well-known that ILD in usual interstitial pneumonitis or non-special interstitial pneumonia pattern frequently locates in lower lung lobes [41]. Our study also showed that the DECT parameters of bilateral lower lung lobes had the highest AUC to differentiate extensive CTD-ILD from the limited CTD-ILD than the ones of middle lobe or upper lobes. The possible reason is that the lesions in lower lung lobes are more serious with more cells, materials or compounds, while DECT is good at detecting and distinguishing them. At the same time, Z_eff_ value and MCTN of middle lung lobe also showed relative higher AUC which corresponded with visual changes in middle lung lobe lesions. The middle lung lobe, with unique ventilation, perfusion and being flanked by two fissures, might affect its specific compliance. It suggested that the distribution of disease should be considered in the severity evaluation of CTD-ILD by DECT.

DECT was indicated to be superior to texture analysis or machine learning methods in view of the degrees of correlation with PFTs in this study. The parameters of DECT significantly associated with FVC% predicted, DLCO, VC, or TLC with correlation coefficients of absolute value ranged from 0.54 to 0.89. In previous studies, the absolute value of correlation coefficients in fibrosis quantitative score based on the texture metrics [17] with FVC% predicted ranged from 0.41 to 0.60, and with DLCO% predicted ranged from 0.37 to 0.68. Combined with ILD patterns, the coefficients of radiologists’ assessment with CALIPER could achieve 0.73, similar to our study. However, all of them need additional software development and long supervised or non-supervised learning. As a novel quantitative technique, DECT with the inherent software is convenient for quantitative analysis in a shorter time. Besides, the DECT quantitative analysis doesn’t need an experienced specialist to define the interstitial patterns. Therefore, DECT seems to have more advantages in evaluating CTD-ILD patients.

The DECT parameters (LV, Z_eff_ value, or MCTN) were associated with the symptoms or life quality of CTD-ILD patients in our study. Respiratory symptoms are the manifestations of structural and inflammatory changes [45] or decline in pulmonary function. The DECT parameters can reflect the material composition and lung structural compliance, and significantly correlated with PFT. That explains why the DECT parameters could differentiate the severity of respiratory symptoms and be their risk factors. Life quality is impaired in ILD patients, influencing clinical prognosis [46]. And SF-36 was recommended to be applied in the comprehensive evaluation of CTD-ILD patients [38]. However, SF-36 was rarely applied in quantitative computed tomography assessment research. In our study, it was indicated that LV, along with age, comorbidity, confirmed CTD and severer cough, contributed to the mental health-related quality of life in CTD-ILD patients. Therefore, DECT could not only complement pulmonary physiology function, but also provide comprehensive clinical features in CTD-ILD patients.

Although MCTN measurement [47, 48] was reported to be relatively unreliable compared to Z_eff_ value, and the precise of measurement may be affected by the presence of surrounding tissues, newer DECT systems, such as the one used in our study have improved accuracy of MCTN with optimized hardware and calculation [49]. Furthermore, MCTN was reported to have highest accuracy near the 70 keV energy level which was selected in our study [50].

While our results were valuable, we recognized there were still several limitations in our study. Firstly, given that there was a significant imbalance in the radiological patterns (usual interstitial pneumonitis and non-special interstitial pneumonia) among the included patients, radiological patterns analysis was not conducted. Secondly, to avoid radiate patients twice, the CT images were reconstructed from DECT data set, thus we could not directly compare CT numbers of conventional CT with MCTN and Z_eff_ value in DECT to document their advantage. Thirdly, the data set was from a single center with a small sample size of a cross sectional study, so it could not be readily generalized. Since our population was ILD patients with CTDs, caution should be exercised in extrapolating our findings to other causes of ILD. We recognized these limitations and believed that further validation of our preliminary results was warranted to predict the progression, acute exacerbation, or death.

## Conclusion

In conclusion, our results suggested that DECT parameters were able to structurally and functionally discriminate severe CTD-ILD patients from mild ones. We believed that DECT could represent an accurate and convenient alternative quantitative tool in evaluating CTD-ILD.

## Supplementary Information

**Additional file 1: Table S1**. Comparison the DECT parameters among TEI>20% and TEI≤20%.**Table S2**. Comparison DECT parameters among Group mild (FVC%≥80% & DLCO%≥80%), Group moderate (the indeterminate) and Group severe (FVC%≤50% or DLCO%≤50%).** Table S3**. Comparison demographic characteristics, volume, Zeff value and monochromatic CTN between limited CTD-ILD/extensive CTD-ILD group and the control group.** Table S4**. Pearson/Spearman correlation coefficient Matrix of DECT parameters with SF-36, TEI and PFT findings.** Table S5**. Receiver operating characteristic curve to demonstrate optimal cutoff value of DECT parameters to detect presence of extensive disease of CTD-ILD.** Table S6**. Comparison the DECT parameters among different degree of dyspnea according to Borg dyspnea score.** Table S7**. Comparison the DECT parameters among different severity cough symptom according to LCQ with the cut-off value 17.** Table S8**. Linear Regression Analysis to Define the Variables Contribution to PCS in patient with CTD-ILD.** Table S9**. Linear Regression Analysis to Define the Variables Contribution to MCS in patient with CTD-ILD.** Table S10**. Binary Logistic Regression Analysis of Univariate Analysis and Multivariate Analysis with Dyspnea of CTD-ILD patient.** Table S11**. Binary Logistic Regression Analysis of Univariate Analysis and Multivariate Analysis with Cough of CTD-ILD patient.

## Data Availability

The datasets used and/or analyzed during the current study are available from the corresponding author on reasonable request.

## Abbreviations

CTD-ILD: Connective tissue disease-associated interstitial lung disease
GSI: Gemstone spectral imaging
CT: Computed tomography
DECT: Dual-energy computed tomography
HRCT: High-resolution computed tomography
LV: Lung volume
Z_eff_: Effective atomic number; Effective-z
MCTN: Monochromatic CT number at 70 keV
HU: Hounsfield units
keV: Kilo electron volt
RA: Rheumatoid arthritis
SSc: Systemic sclerosis/scleroderma
PM/DM: Polymyositis/dermatomyositis
SLE: Systemic lupus erythematosus
SjS: Sjögren syndrome
IPAF: Interstitial pneumonia with autoimmune features
UCTD: Undifferentiated connective tissue disease
SCTD: Specific connective tissue disease
MCTD: Mixed connective tissue disease
RL: Right lower lung lobe
LL: Left lower lung lobe
RM: Right middle lung lobe
RU: Right upper lung lobe
LU: Left upper lung lobe
AUC: Area under the curve
ROC: Receiver operating characteristic
CI: Confidence interval
PCS: Physical component summary
MCS: Mental component summary
