# Supplementary material for: Quantitative evaluation of disease severity in connective tissue disease-associated interstitial lung disease by dual-energy computed tomography
Source: Respir Res. 2022 Mar 5;23:47. doi: 10.1186/s12931-022-01972-4 (PMC8897904; doi:10.1186/s12931-022-01972-4)
Supplement: Supplementary file 1 — Additional file 1: Table S1. Comparison the DECT parameters among TEI>20% and TEI≤20%.Table S2. Comparison DECT parameters among Group mild (FVC%≥80% & DLCO%≥80%), Group moderate (the indeterminate) and Group severe (FVC%≤50% or DLCO%≤50%). Table S3. Comparison demographic characteristics, volume, Zeff value and monochromatic CTN between limited CTD-ILD/extensive CTD-ILD group and the control group. Table S4. Pearson/Spearman correlation coefficient Matrix of DECT parameters with SF-36, TEI and PFT findings. Table S5. Receiver operating characteristic curve to demonstrate optimal cutoff value of DECT parameters to detect presence of extensive disease of CTD-ILD. Table S6. Comparison the DECT parameters among different degree of dyspnea according to Borg dyspnea score. Table S7. Comparison the DECT parameters among different severity cough symptom according to LCQ with the cut-off value 17. Table S8. Linear Regression Analysis to Define the Variables Contribution to PCS in patient with CTD-ILD. Table S9. Linear Regression Analysis to Define the Variables Contribution to MCS in patient with CTD-ILD. Table S10. Binary Logistic Regression Analysis of Univariate Analysis and Multivariate Analysis with Dyspnea of CTD-ILD patient. Table S11. Binary Logistic Regression Analysis of Univariate Analysis and Multivariate Analysis with Cough of CTD-ILD patient. [file 12931_2022_1972_MOESM1_ESM.docx]

TABLE S1. Comparison the DECT parameters among TEI>20% and TEI≤20%.

|  | TEI≤20% | TEI>20% | t /χ^2^ | *p value* |
| --- | --- | --- | --- | --- |
| Total-No. | 85 | 62 |  |  |
| Age (y) | 49.22±9.65 | 51.29±9.57 | -1.287 | *.200* |
| Body mass index(kg/m^2^) | 23.21±3.36 ^§^ | 22.96±3.22 | 0.445 | *.657* |
| Female sex-No. (%) | 73(85.9%) | 59(95.2%) | 3.369 | *.066* |
| Han nationality-No. (%) | 81 (95.3%) | 55(88.7%) | 2.245 | *.203* |
| Former/current smoker-No. (%) | 15(17.6%) | 2(3.2%) | 7.290 | ***.007*** |
| Comorbidity-No. (%) | 55(64.7%) | 32(51.6%) | 2.544 | *.111* |
| Lung Volume (cm^3^) | 3419.03±965.90 | 2563.85±797.25 | 5.697 | ***.000*** |
| V_RL | 613.42±263.28 | 312.01±178.87 | 8.260 | ***.000*** |
| V_RM | 366.34±133.03 | 323.32±149.32 | 1.838 | *.068* |
| V_RU | 883.47±220.66 | 799.63±284.92 | 2.010 | ***.046*** |
| V_LL | 541.44±258.61 | 296.18±166.33 | 6.985 | ***.000*** |
| V_LU | 1014.36±273.74 | 832.72±321.85 | 3.687 | ***.000*** |
| Average Z_eff_ value | 2.211±0.391 | 2.940±0.549 | -8.923 | ***.000*** |
| Z_RL | 2.606±0.561 | 3.663±0.797 | -8.951 | ***.000*** |
| Z_RM | 1.980±0.339 | 2.633±0.649 | -7.231 | ***.000*** |
| Z_RU | 1.885±0.307 | 2.342±0.520 | -6.181 | ***.000*** |
| Z_LL | 2.666±0.625 | 3.611±0.700 | -8.608 | ***.000*** |
| Z_LU | 1.918±0.306 | 2.449±0.543 | -6.932 | ***.000*** |
| Average MCTN (HU) | -807.70±32.81 | -735.71±51.67 | -9.644 | ***.000*** |
| MCTN_RL | -769.12±48.83 | -665.66±77.20 | -9.284 | ***.000*** |
| MCTN_RM | -830.28±28.21 | -766.00±58.82 | -7.963 | ***.000*** |
| MCTN_RU | -839.61±24.50 | -794.05±49.90 | -6.631 | ***.000*** |
| MCTN_LL | -763.14±57.11 | -668.95±69.05 | -8.773 | ***.000*** |
| MCTN_LU | -836.35±24.48 | -783.90±51.07 | -7.484 | ***.000*** |

Total- No.=147, §: N=84.

For abbreviations see Table 1 and 2.

TABLE S2. Comparison DECT parameters among Group mild (FVC%≥80% & DLCO%≥80%), Group moderate (the indeterminate) and Group severe (FVC%≤50% or DLCO%≤50%).

|  |  |  |  |  |  | *P* value | | |
| --- | --- | --- | --- | --- | --- | --- | --- | --- |
|  | Mild | Moderate | Severe | F/χ2 | *P* | Mild *VS* Moderate | Mild *VS* Severe | Moderate *VS* Severe |
| Total-No. | 37 | 57 | 18 |  |  |  |  |  |
| Age (y) | 49.51±7.54 | 50.12±10.37 | 47.83±12.81 | 0.314 | *.732* |  |  |  |
| BMI (kg/m^2^) | 23.88±2.61 | 23.41±3.35 | 21.65±3.73 | 3.070 | *.050* |  |  |  |
| Female-No. (%) | 30(81.1) | 53(93.0) | 17(94.4) | 3.392 | *.174* |  |  |  |
| Han nationality-No. (%) | 35(94.6) | 55(96.5) | 17(94.4) | 0.703 | *.716* |  |  |  |
| Former/current smoker-No. (%) | 9(24.3) | 3(5.3) | 1(5.6) | 7.554 | ***.024*** |  |  |  |
| Comorbidity-No. (%) | 20(54.1) | 37(64.9) | 13(72.2) | 1.994 | *.369* |  |  |  |
| Lung volume (cm^3^) | 3805.52±1136.94 | 3069.00±905.60 | 2458.11±520.96 | 13.715 | ***.000*** | ***.004*** | ***.000*** | ***.002*** |
| V_RL | 732.11±253.94 | 462.91±268.34 | 327.59±149.09 | 20.283 | ***.000*** | ***.000*** | ***.000*** | ***.027*** |
| V_RM | 427.54±153.72 | 343.45±135.99 | 266.02±91.31 | 9.223 | ***.000*** | ***.025*** | ***.000*** | ***.025*** |
| V_RU | 894.65±284.11 | 872.47±268.66 | 835.46±205.31 | 0.303 | *.739* | *.974* | *.761* | *.901* |
| V_LL | 653.32±238.78 | 427.71±247.29 | 249.77±111.60 | 28.182 | ***.000*** | ***.000*** | ***.000*** | ***.000*** |
| V_LU | 1097.91±344.66 | 962.47±307.14 | 779.28±235.19 | 6.522 | ***.002*** | *.158* | ***.001*** | ***.033*** |
| Average Z_eff_ value | 2.186±0.406 | 2.454±0.554 | 2.863±0.570 | 10.664 | ***.000*** | ***.025*** | ***.000*** | ***.036*** |
| Z_RL | 2.531±0.492 | 3.002±0.865 | 3.500±0.804 | 11.221 | ***.000*** | ***.003*** | ***.000*** | *.091* |
| Z_RM | 1.971±0.375 | 2.204±0.557 | 2.567±0.610 | 8.228 | ***.000*** | *.051* | ***.003*** | *.094* |
| Z_RU | 1.926±0.362 | 2.015 ±0.400 | 2.210±0.476 | 3.049 | *.051* | *.597* | *.096* | *.332* |
| Z_LL | 2.568±0.528 | 2.965±0.778 | 3.717±0.818 | 15.289 | ***.000*** | ***.012*** | ***.000*** | ***.006*** |
| Z_LU | 1.936±0.359 | 2.083±0.450 | 2.321±0.555 | 4.630 | ***.012*** | *.228* | ***.038*** | *.291* |
| Average MCTN (HU) | -810.86±33.18 | -782.70±49.90 | -738.84±57.61 | 13.361 | ***.000*** | ***.004*** | ***.000*** | ***.022*** |
| MCTN_RL | -776.95±43.04 | -728.82±80.25 | -679.00±82.98 | 12.119 | ***.000*** | ***.001*** | ***.000*** | *.095* |
| MCTN_RM | -831.84±31.64 | -807.63±48.03 | -767.28±60.98 | 10.304 | ***.000*** | ***.012*** | ***.001*** | *.049* |
| MCTN_RU | -836.38±29.34 | -825.53±37.14 | -803.50±47.19 | 4.884 | ***.009*** | *.314* | ***.036*** | *.222* |
| MCTN_LL | -773.76±42.87 | -732.05±74.49 | -652.44±81.88 | 18.610 | ***.000*** | ***.003*** | ***.000*** | ***.003*** |
| MCTN_LU | -835.41±28.20 | -819.47±39.24 | -792.00±55.47 | 5.915 | ***.006*** | *.071* | ***.015*** | *.174* |

Total No.=112.

For abbreviations see Table 1 and 2.

TABLE S3: Comparison demographic characteristics, volume, Z_eff_ value and monochromatic CTN between limited CTD-ILD/extensive CTD-ILD group and the control group.

|  | Control | Limited ILD | Extensive ILD | F/χ^2^ |  | *P value* | | | *P value*  *for trend* |
| --- | --- | --- | --- | --- | --- | --- | --- | --- | --- |
|  |  |  |  |  | *p* | *C vs L* | *C vs E* | *L vs E* |  |
| Total-No. | 32 | 89 | 39 |  |  |  |  |  |  |
| Age (y) | 49.53±8.15 | 49.44±9.27 | 51.64±11.55 | 0.749 | *.475* |  |  |  |  |
| BMI (kg/m^2^) | 23.78±4.26 | 23.49±3.09 | 22.53±3.64 | 1.368 | *.258* |  |  |  |  |
| Female sex-No. (%) | 27(84.4%) | 78(87.6%) | 38(97.4%) | 4.063 | *.117* |  |  |  |  |
| Han nationality-No. (%) | 29(90.6%) | 86 (96.6%) | 35(89.7%) | 3.243 | *.194* |  |  |  |  |
| Former/current smoker-No. (%) | 3(9.4%) | 14(15.7%) | 0(0) | 8.113 | ***.013*** |  |  |  |  |
| Lung volume (cm^3^) | 3761.63±1243.86 | 3475.21±971.62 | 2309.51±668.98 | 23.751 | ***.000*** | *.154* | ***.000*** | ***.000*** | ***.000*** |
| V_RL | 895.38±346.64 | 607.08±275.18 | 278.39±136.74 | 47.212 | ***.000*** | ***.000*** | ***.000*** | ***.000*** | ***.000*** |
| V_RM | 374.74±123.27 | 377.19±138.00 | 287.47±144.82 | 6.244 | ***.002*** | *.931* | ***.008*** | ***.001*** | ***.008*** |
| V_RU | 751.32±222.75 | 901.38±249.48 | 742.69±262.74 | 7.718 | ***.001*** | ***.004*** | *.884* | ***.001*** | *.884* |
| V_LL | 805.86±328.73 | 548.31±255.13 | 254.51±120.13 | 42.965 | ***.000*** | ***.000*** | ***.000*** | ***.000*** | ***.000*** |
| V_LU | 934.33±300.42 | 1041.24±284.10 | 746.45±316.93 | 13.529 | ***.000*** | *.081* | ***.009*** | ***.000*** | ***.009*** |
| Average Z_eff_ value | 2.417±0.779 | 2.256±0.435 | 3.104±0.569 | 24.284 | ***.000*** | *.159* | ***.000*** | ***.000*** | ***.000*** |
| Z_RL | 2.622±0.892 | 2.682±0.653 | 3.828±0.813 | 30.143 | ***.000*** | *.695* | ***.000*** | ***.000*** | ***.000*** |
| Z_RM | 2.219±0.679 | 2.018±0.382 | 2.833±0.692 | 22.697 | ***.000*** | *.071* | ***.000*** | ***.000*** | ***.000*** |
| Z_RU | 2.248±0.651 | 1.911±0.327 | 2.501±0.567 | 15.980 | ***.000*** | ***.001*** | ***.025*** | ***.000*** | ***.025*** |
| Z_LL | 2.706±0.987 | 2.706±0.663 | 3.792±0.698 | 25.920 | ***.000*** | *.998* | ***.000*** | ***.000*** | ***.000*** |
| Z_LU | 2.288±0.737 | 1.961±0.334 | 2.566±0.629 | 13.333 | ***.000*** | ***.002*** | ***.025*** | ***.000*** | ***.025*** |
| Average MCTN(HU) | -805.94±48.52 | -802.20±40.27 | -722.87±52.83 | 40.438 | ***.000*** | *.689* | ***.000*** | ***.000*** | ***.000*** |
| MCTN_RL | -789.72±58.32 | -760.08±63.10 | -654.62±75.83 | 46.483 | ***.000*** | ***.030*** | ***.000*** | ***.000*** | ***.000*** |
| MCTN_RM | -825.25±41.42 | -825.56±35.10 | -749.33±61.82 | 35.562 | ***.000*** | *.973* | ***.000*** | ***.000*** | ***.000*** |
| MCTN_RU | -822.25±40.33 | -836.07±29.22 | -781.03±54.10 | 21.157 | ***.000*** | *.086* | ***.000*** | ***.000*** | ***.000*** |
| MCTN_LL | -782.69±64.52 | -757.79±63.46 | -654.72±69.34 | 43.511 | ***.000*** | *.066* | ***.000*** | ***.000*** | ***.000*** |
| MCTN_LU | -809.81±64.56 | -831.51±28.42 | -774.64±60.19 | 13.706 | ***.000*** | ***.025*** | ***.002*** | ***.000*** | ***.002*** |

For abbreviations see Table 1 and 2.

Table S4. Pearson/Spearman correlation coefficient Matrix of DECT parameters with SF-36, TEI and PFT findings.

|  |  | Lung volume(cm^3^) | Average Z_eff_ value | Average MCTN(HU) | V_RL | V_RM | V_RU | V_LL | V_LU | Z_RL | Z_RM | Z_RU | Z_LL | Z_LU | MCTN  _RL | MCTN  _RM | MCTN  _RU | MCTN  _LL | MCTN  _LU |
| --- | --- | --- | --- | --- | --- | --- | --- | --- | --- | --- | --- | --- | --- | --- | --- | --- | --- | --- | --- |
| PCS | r | .231^**^ | -.271^**^ | -.283^**^ | .182^*^ | .087 | .137 | .210^*^ | .256^**^ | -.182^*^ | -.265^**^ | -.260^**^ | -.262^**^ | -.309^**^ | -.181^*^ | -.281^**^ | -.277^**^ | -.268^**^ | -.328^**^ |
|  | N | 145 | 145 | 145 | 145 | 145 | 145 | 145 | 145 | 145 | 145 | 145 | 145 | 145 | 145 | 145 | 145 | 145 | 145 |
| MCS | r | .187^*^ | -.113 | -.105 | .172^*^ | .053 | .205^*^ | .111 | .164^*^ | -.100 | -.097 | -.178^*^ | -.038 | -.152 | -.093 | -.093 | -.168^*^ | -.031 | -.145 |
|  | N | 145 | 145 | 145 | 145 | 145 | 145 | 145 | 145 | 145 | 145 | 145 | 145 | 145 | 145 | 145 | 145 | 145 | 145 |
| no/mild/severe dyspnea | rho | -.302^**^ | .291^**^ | .295^**^ | -.235^**^ | -.064 | -.187^*^ | -.285^**^ | -.269^**^ | .243^**^ | .210^*^ | .260^**^ | .313^**^ | .293^**^ | .253^**^ | .211^*^ | .270^**^ | .305^**^ | .308^**^ |
|  | N | 145 | 145 | 145 | 145 | 145 | 145 | 145 | 145 | 145 | 145 | 145 | 145 | 145 | 145 | 145 | 145 | 145 | 145 |
| no/mild/severe cough | rho | -.192^*^ | .174^*^ | .153 | -.199^*^ | .008 | -.080 | -.209^*^ | -.140 | .208^*^ | .116 | .165^*^ | .166^*^ | .160 | .182^*^ | .100 | .145 | .149 | .141 |
|  | N | 145 | 145 | 145 | 145 | 145 | 145 | 145 | 145 | 145 | 145 | 145 | 145 | 145 | 145 | 145 | 145 | 145 | 145 |
| TEI (%) | r | -.485^**^ | .712^**^ | .742^**^ | -.529^**^ | -.237^**^ | -.218^**^ | -.490^**^ | -.396^**^ | .630^**^ | .663^**^ | .648^**^ | .637^**^ | .702^**^ | .640^**^ | .702^**^ | .679^**^ | .647^**^ | .743^**^ |
|  | N | 147 | 147 | 147 | 147 | 147 | 147 | 147 | 147 | 147 | 147 | 147 | 147 | 147 | 147 | 147 | 147 | 147 | 147 |
| FVC(L) | r | .890^**^ | -.533^**^ | -.561^**^ | .800^**^ | .648^**^ | .539^**^ | .810^**^ | .766^**^ | -.479^**^ | -.529^**^ | -.462^**^ | -.486^**^ | -.490^**^ | -.487^**^ | -.567^**^ | -.496^**^ | -.488^**^ | -.534^**^ |
|  | N | 114 | 114 | 114 | 114 | 114 | 114 | 114 | 114 | 114 | 114 | 114 | 114 | 114 | 114 | 114 | 114 | 114 | 114 |
| FVC%predicted | r | .667^**^ | -.542^**^ | -.582^**^ | .668^**^ | .463^**^ | .312^**^ | .690^**^ | .530^**^ | -.510^**^ | -.506^**^ | -.443^**^ | -.527^**^ | -.466^**^ | -.531^**^ | -.551^**^ | -.485^**^ | -.540^**^ | -.519^**^ |
|  | N | 114 | 114 | 114 | 114 | 114 | 114 | 114 | 114 | 114 | 114 | 114 | 114 | 114 | 114 | 114 | 114 | 114 | 114 |
| FEV1%predicted | r | .580^**^ | -.516^**^ | -.558^**^ | .613^**^ | .380^**^ | .226^*^ | .647^**^ | .439^**^ | -.504^**^ | -.476^**^ | -.410^**^ | -.499^**^ | -.429^**^ | -.528^**^ | -.521^**^ | -.454^**^ | -.517^**^ | -.481^**^ |
|  | N | 114 | 114 | 114 | 114 | 114 | 114 | 114 | 114 | 114 | 114 | 114 | 114 | 114 | 114 | 114 | 114 | 114 | 114 |
| FEV1%FVC | r | -.369^**^ | .152 | .155 | -.243^**^ | -.264^**^ | -.325^**^ | -.244^**^ | -.390^**^ | .083 | .136 | .163 | .158 | .188^*^ | .082 | .144 | .171 | .150 | .204^*^ |
|  | N | 114 | 114 | 114 | 114 | 114 | 114 | 114 | 114 | 114 | 114 | 114 | 114 | 114 | 114 | 114 | 114 | 114 | 114 |
| VC(L) | r | .892^**^ | -.538^**^ | -.565^**^ | .802^**^ | .648^**^ | .541^**^ | .813^**^ | .767^**^ | -.484^**^ | -.533^**^ | -.466^**^ | -.490^**^ | -.495^**^ | -.491^**^ | -.571^**^ | -.500^**^ | -.492^**^ | -.539^**^ |
|  | N | 114 | 114 | 114 | 114 | 114 | 114 | 114 | 114 | 114 | 114 | 114 | 114 | 114 | 114 | 114 | 114 | 114 | 114 |
| VC%predicted | r | .667^**^ | -.555^**^ | -.595^**^ | .673^**^ | .463^**^ | .303^**^ | .695^**^ | .527^**^ | -.523^**^ | -.520^**^ | -.452^**^ | -.539^**^ | -.476^**^ | -.544^**^ | -.566^**^ | -.495^**^ | -.552^**^ | -.529^**^ |
|  | N | 114 | 114 | 114 | 114 | 114 | 114 | 114 | 114 | 114 | 114 | 114 | 114 | 114 | 114 | 114 | 114 | 114 | 114 |
| TLC(L) | r | .872** | -.501** | -.528** | .749** | .620** | .582** | .790** | .747** | -.455** | -.475** | -.436** | -.472** | -.453** | -.465** | -.509** | -.211^*^ | -.215^*^ | -.215^*^ |
|  | N | 114 | 114 | 114 | 114 | 114 | 114 | 114 | 114 | 114 | 114 | 114 | 114 | 114 | 114 | 114 | 114 | 114 | 114 |
| TLC%predicted | r | .704** | -.581** | -.616** | .693** | .499** | .353** | .735** | .538** | -.559** | -.538** | -.467** | -.573** | -.471** | -.576** | -.580** | -.507^**^ | -.581^**^ | -.518^**^ |
|  | N | 114 | 114 | 114 | 114 | 114 | 114 | 114 | 114 | 114 | 114 | 114 | 114 | 114 | 114 | 114 | 114 | 114 | 114 |
| DLCO(ml/min/  mmHg) | r | .699^**^ | -.481^**^ | -.550^**^ | .721^**^ | .541^**^ | .298^**^ | .738^**^ | .519^**^ | -.483^**^ | -.448^**^ | -.319^**^ | -.511^**^ | -.348^**^ | -.520^**^ | -.522^**^ | -.393^**^ | -.559^**^ | -.430^**^ |
|  | N | 112 | 112 | 112 | 112 | 112 | 112 | 112 | 112 | 112 | 112 | 112 | 112 | 112 | 112 | 112 | 112 | 112 | 112 |
| DlCO%predicted | r | .397^**^ | -.371^**^ | -.427^**^ | .459^**^ | .368^**^ | .058 | .534^**^ | .219^*^ | -.412^**^ | -.300^**^ | -.176 | -.452^**^ | -.210^*^ | -.443^**^ | -.357^**^ | -.233^*^ | -.496^**^ | -.273^**^ |
|  | N | 112 | 112 | 112 | 112 | 112 | 112 | 112 | 112 | 112 | 112 | 112 | 112 | 112 | 112 | 112 | 112 | 112 | 112 |

a According to the Borg dyspnea score. b According to the Leicester cough questionnaire (LCQ). **p<0.01, *P<0.05

For abbreviations see Table 1 and 2. TABLE S5. Receiver operating characteristic curve to demonstrate optimal cutoff value of DECT parameters to detect presence of extensive disease of CTD-ILD.

|  | **AUC (95%CI)** | **Sensitivity** | **Specificity** | **Youden Index** | **Cutoff Value** | ***P value*** |
| --- | --- | --- | --- | --- | --- | --- |
| Average Z_eff_ value | 0.889 (0.835-0.943) | 87.2% | 76.4% | 0.636 | 2.510 | *.000* |
| Z_RL | 0.878 (0.821-0.936) | 97.4% | 65.2% | 0.626 | 2.775 | *.000* |
| Z_RM | 0.864 (0.792-0.935) | 71.8% | 91.0% | 0.628 | 2.537 | *.000* |
| Z_RU | 0.823 (0.743-0.903) | 79.5% | 80.9% | 0.604 | 2.091 | *.000* |
| Z_LL | 0.872 (0.811-0.932) | 84.6% | 78.7% | 0.633 | 3.174 | *.000* |
| Z_LU | 0.809 (0.720-0.898) | 79.5% | 76.4% | 0.559 | 2.156 | *.000* |
| Average MCTN (HU) | 0.901 (0.850-0.952) | 82.1% | 85.4% | 0.674 | -762.30 | *.000* |
| MCTN_RL | 0.879(0.821-0.936) | 94.9% | 70.8% | 0.657 | -746.50 | *.000* |
| MCTN_RM | 0.876 (0.807-0.945) | 82.1% | 82.0% | 0.641 | -798.50 | *.000* |
| MCTN_RU | 0.829 (0.750-0.909) | 76.9% | 85.4% | 0.623 | -815.00 | *.000* |
| MCTN_LL | 0.872 (0.812-0.932) | 92.3% | 70.8% | 0.631 | -743.50 | *.000* |
| MCTN_LU | 0.817 (0.728-0.905) | 74.4% | 79.8% | 0.541 | -808.50 | *.000* |
| Lung volume (cm^3^) | 0.121 (0.055-0.187) | 7.7% | 27.0% | -0.653 | 2903.60 | *.000* |
| V_RL | 0.137 (0.076-0.199) | 15.4% | 25.8% | -0.588 | 410.91 | *.000* |
| V_RM | 0.297 (0.194-0.400) | 41.0% | 20.2% | -0.387 | 279.95 | *.000* |
| V_RU | 0.271 (0.174-0.368) | 38.5% | 18.0% | -0.436 | 722.75 | *.000* |
| V_LL | 0.143 (0.079-0.206) | 17.9% | 23.6% | -0.585 | 355.47 | *.000* |
| V_LU | 0.182 (0.098-0.265) | 33.3% | 11.2% | -0.554 | 729.51 | *.000* |

AUC, area under curve; ROC, Receiver operating characteristic; CTD-ILD, connective tissue associated interstitial lung disease.

For abbreviations see Table 1 and 2.

TABLE S6. Comparison the DECT parameters among different degree of dyspnea according to Borg dyspnea score.

|  | No dyspnea | Mild dyspnea | Moderate to severe dyspnea |  | *p* | *p value* | | |
| --- | --- | --- | --- | --- | --- | --- | --- | --- |
|  |  |  |  | F/χ^2^ |  | *0 vs 1* | *0 vs 2* | *1 vs 2* |
| Total-No. | 76 | 53 | 16 |  |  |  |  |  |
| Age (y) | 50.93±9.17 | 48.70±9.51 | 50.44±11.39 | 0.869 | *.422* |  |  |  |
| BMI (kg/m^2^) | 22.76±3.23 | 23.69±3.46 | 22.57±2.80 | 1.493 | *.228* |  |  |  |
| Female-No. (%) | 64(84.2) | 50(94.3) | 16(100) | 5.529 | .*063* |  |  |  |
| Han nationality-No. (%) | 70(92.1) | 49(92.5) | 15(93.8) | 0.125 | *1.000* |  |  |  |
| Former/current smoker-No. (%) | 12 (15.8) | 5(9.4) | 0(0) | 3.607 | *.165* |  |  |  |
| Comorbidity- No. (%) | 44(57.9) | 29(54.7) | 12(75.0) | 2.119 | *.347* |  |  |  |
| Lung volume (cm^3^) | 3260.06±909.29 | 2939.26±1088.27 | 2525.42±849.43 | 4.430 | ***.014*** | *.067* | ***.007*** | *.138* |
| V_RLL | 531.39±264.08 | 465.55±290.32 | 343.08±242.11 | 3.421 | ***.035*** | *.178* | ***.013*** | *.116* |
| V_RML | 349.86±137.06 | 357.32±149.24 | 300.13±139.30 | 1.029 | *.360* | *.769* | *.205* | *.160* |
| V_RUL | 885.89±247.95 | 817.52±263.39 | 777.38±234.85 | 1.868 | *.158* | *.132* | *.120* | *.578* |
| V_LLL | 489.91±243.08 | 407.39±270.33 | 302.28±190.44 | 4.442 | ***.013*** | *.066* | ***.007*** | *.141* |
| V_LUL | 1003.00±285.97 | 891.49±337.16 | 802.55±250.09 | 3.985 | ***.021*** | ***.041*** | ***.017*** | *.304* |
| Average Z_eff_ value | 2.357±0.467 | 2.658±0.660 | 2.854±0.649 | 6.356 | ***.003*** | ***.003*** | ***.002*** | *.226* |
| Z_RLL | 2.857±0.729 | 3.240±0.936 | 3.385±0.932 | 4.721 | ***.010*** | ***.011*** | ***.022*** | *.542* |
| Z_RML | 2.137±0.478 | 2.336±0.650 | 2.600±0.725 | 3.924 | ***.027*** | *.055* | ***.004*** | *.110* |
| Z_RUL | 1.960±0.362 | 2.193±0.553 | 2.284±0.491 | 5.183 | ***.009*** | ***.005*** | ***.011*** | *.485* |
| Z_LLL | 2.834±0.679 | 3.248±0.840 | 3.573±0.937 | 8.387 | ***.000*** | ***.003*** | ***.001*** | *.142* |
| Z_LUL | 1.999±0.327 | 2.271±0.614 | 2.427±0.542 | 6.783 | ***.002*** | ***.002*** | ***.001*** | *.251* |
| Average MCTN (HU) | -792.14±43.80 | -767.04±59.63 | -738.71±64.93 | 6.665 | ***.003*** | ***.009*** | ***.000*** | *.061* |
| MCTN_RLL | -743.83±69.91 | -710.55±86.07 | -685.56±93.16 | 4.265 | ***.020*** | ***.020*** | ***.008*** | *.268* |
| MCTN_RML | -813.54±45.87 | -798.96±54.34 | -763.94±72.49 | 4.465 | ***.018*** | *.122* | ***.001*** | ***.021*** |
| MCTN_RUL | -830.89±34.85 | -812.06±49.48 | -796.13±48.73 | 5.061 | ***.010*** | ***.014*** | ***.003*** | *.189* |
| MCTN_LLL | -745.34±62.60 | -708.64±82.53 | -666.75±93.70 | 7.107 | ***.002*** | ***.006*** | ***.000*** | ***.049*** |
| MCTN_LUL | -827.08±30.62 | -805.00±55.03 | -781.19±54.36 | 6.908 | ***.002*** | ***.006*** | ***.000*** | *.059* |

Total-No.=145.

BMI: body mass index;

No dyspnea(0): Borg dyspnea scale=0; Mild dyspnea(1): 0 < Borg dyspnea scale ≤ 2; Moderate to severe dyspnea(2): Borg dyspnea scale≥3

TABLE S7. Comparison the DECT parameters among different severity cough symptom according to LCQ with the cut-off value 17.

|  | No cough | Mild cough | Severe cough |  | *p* | *p value* | | |
| --- | --- | --- | --- | --- | --- | --- | --- | --- |
|  |  |  |  | F/χ^2^ |  | *0 vs 1* | *0 vs 2* | *1 vs 2* |
| Total-No. | 81 | 39 | 25 |  |  |  |  |  |
| Age (y) | 51.36 ±8.65 | 49.44±9.46 | 46.84±11.76 | 2.295 | *.105* |  |  |  |
| BMI (kg/m^2^) | 23.20±2.97 | 22.90±3.53 | 22.97±3.95 | 0.121 | *.887* |  |  |  |
| Female-No. (%) | 72(88.9) | 37(94.9) | 21(84.0) | 2.103 | *.338* |  |  |  |
| Han nationality-No. (%) | 72(88.9) | 38 (97.4) | 24(96.0) | 2.706 | *.240* |  |  |  |
| Former/current smoker-No. (%) | 10 (12.3) | 2(5.1) | 5(20.0) | 3.308 | *.192* |  |  |  |
| Comorbidity-No. (%) | 46(56.8) | 25(64.1) | 14(56.0) | 0.666 | *.717* |  |  |  |
| Lung volume (cm^3^) | 3222.62±981.03 | 2758.39±1011.19 | 3013.70±940.15 | 2.975 | *.054* | ***.017*** | *.354* | *.312* |
| V_RLL | 532.55±279.89 | 425.40±279.15 | 432.88±239.83 | 2.605 | *.077* | *.046* | *.113* | *.915* |
| V_RML | 350.46±133.28 | 331.12±160.38 | 361.13±142.19 | .389 | *.679* | *.487* | *.744* | *.413* |
| V_RUL | 871.97±267.03 | 797.81±220.61 | 854.01±256.92 | 1.131 | *.326* | *.136* | *.757* | *.389* |
| V_LLL | 485.77±248.35 | 353.31±263.49 | 421.40±231.02 | 3.780 | ***.025*** | ***.007*** | *.262* | *.289* |
| V_LUL | 981.87±298.77 | 850.75±311.64 | 944.27±317.96 | 2.426 | *.092* | ***.029*** | *.592* | *.234* |
| Average Z_eff_ value | 2.408±0.505 | 2.672±0.680 | 2.656±0.638 | 3.534 | ***.032*** | ***.021*** | *.064* | *.914* |
| Z_RLL | 2.888±0.760 | 3.242±0.938 | 3.304±0.917 | 3.666 | ***.028*** | ***.032*** | ***.032*** | *.773* |
| Z_RML | 2.169±0.467 | 2.397±0.777 | 2.344±0.588 | 1.922 | *.153* | ***.047*** | *.194* | *.721* |
| Z_RUL | 1.997±0.386 | 2.185±0.553 | 2.191±0.536 | 3.023 | *.052* | ***.039*** | *.069* | *.960* |
| Z_LLL | 2.923±0.747 | 3.291±0.885 | 3.184±0.815 | 3.128 | ***.047*** | ***.019*** | *.155* | *.601* |
| Z_LUL | 2.063±0.412 | 2.245±0.577 | 2.257±0.586 | 2.566 | *.080* | *.060* | *.088* | *.925* |
| Average MCTN (HU) | -787.55±47.33 | -759.46±62.75 | -770.58±60.31 | 3.207 | ***.046*** | ***.009*** | *.173* | *.424* |
| MCTN_RLL | -740.05±74.46 | -703.15±86.55 | -711.68±85.46 | 3.252 | ***.042*** | ***.019*** | *.122* | *.677* |
| MCTN_RML | -811.01±43.38 | -787.05±71.11 | -800.40±52.39 | 2.248 | *.112* | ***.023*** | *.389* | *.333* |
| MCTN_RUL | -828.20±34.01 | -807.33±52.99 | -814.20±51.53 | 2.633 | *.079* | ***.014*** | *.157* | *.534* |
| MCTN_LLL | -736.98±71.63 | -697.82±85.03 | -718.48±79.85 | 3.477 | ***.034*** | ***.010*** | *.295* | *.296* |
| MCTN_LUL | -821.53±37.24 | -801.92±53.70 | -808.12±55.91 | 2.678 | *.072* | ***.029*** | *.201* | *.597* |

Total-No.=145.

BMI: body mass index;

No cough: LCQ score=0; Mild cough: LCQ score ≥17; Moderate to severe cough: 0 < LCQ score < 17

TABLE S8. Linear Regression Analysis to Define the Variables Contribution to PCS in patient with CTD-ILD.

| Characteristic | Unadjusted (n=145) | | | | Adjusted ^a^ (n=145) | | | |
| --- | --- | --- | --- | --- | --- | --- | --- | --- |
|  | β value | |  |  | β value | |  |  |
|  | Unstandardized | standardized | 95%CI | *P* | Unstandardized | standardized | 95%CI | *P* |
| Age (y) | -0.094 | -0.103 | -0.244, -0.056 | *.218* | -0.149 | -0.164 | -0.277, -0.021 | ***.023*** |
| Gender (male/female) | -2.556 | -0.090 | -7.249, 2.137 | *.283* |  |  |  |  |
| Han nationality | 0.820 | 0.025 | -4.597, 6.238 | *.765* |  |  |  |  |
| BMI (kg/m^2^) | -0.101 | -0.038 | -0.539, 0.336 | *.648* |  |  |  |  |
| Former/current smoke | 1.046 | 0.039 | -3.411, 5.503 | *.643* |  |  |  |  |
| Comorbidity | -2.841 | -0.161 | -5.717, 0.034 | *.053* | -2.231 | -0.127 | -4.668, 0.206 | *.072* |
| Lung volume (L) | 2.019 | 0.231 | 0.613, 3.426 | ***.005*** |  |  |  |  |
| Average Z_eff_ value | -4.000 | -0.271 | -6.348, -1.651 | ***.001*** |  |  |  |  |
| Average MCTN (HU) | -0.045 | -0.283 | -0.070, -0.020 | ***.001*** |  |  |  |  |
| Type of CTD (UCTD/SCTD/MCTD) | -0.480 | -0.030 | -3.100, 2.140 | *.718* |  |  |  |  |
| Dyspnea (no/mild/severe) ^b^ | -6.461 | -0.507 | -8.278, -4.643 | ***.000*** | -6.240 | -0.489 | -8.017, -4.462 | ***.000*** |
| Cough (no/mild/severe) ^c^ | -2.246 | -0.197 | -4.090, -0.402 | ***.017*** | -1.899 | -0.167 | -3.504, -0.295 | ***.021*** |

^a^ Adjusted for age, gender, BMI, former/current smoke and variables listed above. ^b^ According to the Borg dyspnea score. ^c^ According to the Leicester cough questionnaire (LCQ). Significance with p<0.05.

CTD-ILD connective tissue disease associated interstitial lung disease, PCS Physical Component Summary, BMI body mass index, MCTN monochromatic CT Number at 70 keV, CTD connective tissue disease, UCTD Undifferentiated CTD, SCTD Specific CTD, MCTD mixed CTD.

TABLE S9. Linear Regression Analysis to Define the Variables Contribution to MCS in patient with CTD-ILD.

| Characteristic | Unadjusted (n=145) | | | | Adjusted^a^ (n=145) | | | |
| --- | --- | --- | --- | --- | --- | --- | --- | --- |
|  | β value | |  |  | β value | |  |  |
|  | Unstandardized | standardized | 95%CI | *P* | Unstandardized | standardized | 95%CI | *P* |
| Age (y) | 0.253 | 0.228 | 0.075, 0.431 | ***.006*** | 0.160 | 0.145 | -0.021, 0.342 | *.083* |
| Gender (male/female) | -4.468 | -0.129 | -10.135, 1.199 | *.121* |  |  |  |  |
| Han nationality | -2.033 | -0.051 | -8.598, 4.532 | *.541* |  |  |  |  |
| BMI (kg/m^2^) | -0.029 | -0.009 | -0.560, 0.502 | *.914* |  |  |  |  |
| Former/current smoke | 3.557 | 0.109 | -1.821, 8.935 | *.193* |  |  |  |  |
| Comorbidity | 3.669 | 0.172 | 0.188, 7.151 | ***.039*** | 4.168 | 0.195 | 0.948, 7.388 | ***.012*** |
| Lung volume (L) | 1.982 | 0. 187 | 0.259, 3.705 | ***.024*** | 2.101 | 0.198 | 0.470, 3.732 | ***.012*** |
| Average Z_eff_ value | -2.017 | -0.113 | -4.958, 0.924 | *.177* |  |  |  |  |
| Average MCTN | -0.020 | -0.105 | -0.052, -0.011 | *.208* |  |  |  |  |
| Type of CTD (UCTD/SCTD/MCTD) | -4.437 | -0.231 | -7.531, -1.343 | ***.005*** | -4.166 | -0.217 | -7.278, -1.053 | ***.009*** |
| Dyspnea (no/mild/severe) ^b^ | -1.847 | -0.119 | -4.386, 0.692 | *.153* |  |  |  |  |
| Cough (no/mild/severe) ^c^ | -3.188 | -0.231 | -5.408, -0.968 | ***.005*** | -2.956 | -0.214 | -5.135, -0.777 | ***.008*** |

^a^ Adjusted for age, gender, BMI, former/current smoke and variables listed above. b According to the Borg dyspnea score. c According to the Leicester cough questionnaire (LCQ). Significance with p<0.05.

CTD-ILD connective tissue disease associated interstitial lung disease, MCS Mental Component Summary, BMI body mass index, MCTN monochromatic CT Number at 70 keV, CTD connective tissue disease, UCTD Undifferentiated CTD, SCTD Specific CTD, MCTD mixed CTD.

TABLE S10. Binary Logistic Regression Analysis of Univariate Analysis and Multivariate Analysis with Dyspnea of CTD-ILD patient.

|  | Univariate Analysis | | | | | Multivariate Analysis | | | | |
| --- | --- | --- | --- | --- | --- | --- | --- | --- | --- | --- |
|  | β value | SE | Wals | OR (95%CI) | *P* | β value | SE | Wals | OR (95%CI) | *P* |
| Age (y) | -0.020 | 0.018 | 1.328 | 0.980(0.946-1.014) | *.249* | -0.040 | 0.020 | 4.276 | 0.960(0.924- 0.998) | **.039** |
| Gender (male/female) | 1.417 | 0.669 | 4.488 | 4.125(1.112-15.304) | ***.034*** |  |  |  |  |  |
| Han nationality | 0.093 | 0.630 | 0.022 | 1.097(0.319-3.770) | *.883* |  |  |  |  |  |
| BMI (kg/m^2^) | 0.063 | 0.051 | 1.516 | 1.065(0.963-1.179) | *.218* |  |  |  |  |  |
| Former/current smoke | -0.875 | 0.561 | 2.436 | 0.417(0.139-1.251) | *.119* |  |  |  |  |  |
| Comorbidity | 0.063 | 0.338 | 0.035 | 1.065(0.549-2.065) | *.852* |  |  |  |  |  |
| Lung volume (L) | -0.461 | 0.188 | 5.982 | 0.631(0.436-0.913) | ***.014*** |  |  |  |  |  |
| Average Z_eff_ value | 1.096 | 0.325 | 11.379 | 2.991(1.583-5.653) | ***.001*** |  |  |  |  |  |
| Average MCTN (HU) | 0.011 | 0.003 | 10.888 | 1.012(1.005-1.018) | ***.001*** | 0.014 | 0.004 | 13.108 | 1.014 (1.006- 1.021) | ***.000*** |
| Type of CTD |  |  | 2.398 |  | *.302* |  |  |  |  |  |
| SCTD vs UCTD | 0.507 | 0.428 | 1.398 | 1.660(0.717-3.843) | *.237* |  |  |  |  |  |
| MCTD vs UCTD | 0.952 | 0.649 | 2.151 | 2.591(0.726-9.246) | *.142* |  |  |  |  |  |
| Note: N=145, significance with p<0.05  CTD-ILD connective tissue disease associated interstitial lung disease, BMI body mass index, MCTN monochromatic CT number at 70 keV, CTD connective tissue disease, SCTD specific connective tissue disease, UCTD undifferentiated connective tissue disease, MCTD mixed connective tissue disease. | | | | | | | | | | |

TABLE S11. Binary Logistic Regression Analysis of Univariate Analysis and Multivariate Analysis with Cough of CTD-ILD patient.

|  | Univariate Analysis | | | | | Multivariate Analysis | | | | |
| --- | --- | --- | --- | --- | --- | --- | --- | --- | --- | --- |
|  | β value | SE | Wals | OR (95%CI) | *P* | β value | SE | Wals | OR (95%CI) | *P* |
| Age (y) | -0.033 | 0.018 | 3.316 | 0.967(0.933-1.003) | *.069* | -0.074 | 0.023 | 10.136 | 0.929(0.888-0.972) | ***.001*** |
| Gender (male/female) | 0.189 | 0.556 | 0.116 | 1.208(0.407-3.592) | *.733* |  |  |  |  |  |
| Han nationality | 1.355 | 0.801 | 2.862 | 3.875(0.807-18.614) | *.091* | 1.490 | 0.870 | 2.930 | 4.435(0.806-24.413) | *.087* |
| BMI (kg/m^2^) | -0.025 | 0.051 | 0.238 | 0.975(0.882-1.078) | *.626* |  |  |  |  |  |
| Former/current smoke | -0.137 | 0.524 | 0.068 | 0.872(0.312-2.435) | *.794* |  |  |  |  |  |
| Comorbidity | 0.171 | 0.341 | 0.253 | 1.187(0.609-2.314) | *.615* |  |  |  |  |  |
| Lung volume (L) | -0.401 | 0.187 | 4.595 | 0.670(0.464-0.966) | ***.032*** |  |  |  |  |  |
| Average Z_eff_ value | 0.771 | 0.302 | 6.504 | 2.161(1.195-3.907) | ***.011*** |  |  |  |  |  |
| Average MCTN (HU) | 0.008 | 0.003 | 6.318 | 1.008(1.002-1.015) | ***.012*** | 0.012 | 0.004 | 9.223 | 1.012(1.004- 1.019) | ***.002*** |
| Type of CTD |  |  | 4.909 |  | *.086* |  |  | 10.516 |  | ***.005*** |
| SCTD vs UCTD | -0.728 | 0.424 | 2.947 | 0.483(0.210-1.109) | *.086* | -1.377 | 0.511 | 7.269 | 0.252(0.093-0.687) | ***.007*** |
| MCTD vs UCTD | -1.417 | 0.693 | 4.185 | 0.242(0.062-0.942) | ***.041*** | -2.457 | 0.819 | 8.990 | 0.086(0.017-0.427) | ***.003*** |
| Note: N=145, significance with p<0.05  CTD-ILD connective tissue disease associated interstitial lung disease, BMI body mass index, MCTN monochromatic CT Number at 70 keV, CTD connective tissue disease, SCTD specific connective tissue disease, UCTD undifferentiated connective tissue disease, MCTD mixed connective tissue disease. | | | | | | | | | | |
